# Factors related to under-diagnosis and under-treatment of childhood asthma in metropolitan France

**DOI:** 10.1186/2049-6958-7-24

**Published:** 2012-08-08

**Authors:** Isabella Annesi-Maesano, Carla Sterlin, Denis Caillaud, Fréderic de Blay, François Lavaud, Denis Charpin, Chantal Raherisson

**Affiliations:** 1 U707, EPAR, INSERM, Paris, France; 2 EPAR, UMR-s 707, UPMC Paris Sorbonnes, Paris, France; 3 University of Usherbrooke, Usherbrooke, Canada; 4 Service de Pneumologie et Allergologie, CHU, Clermont-Ferrand, France; 5 Service de Pneumologie et Allergologie, CHU, Strasbourg, France; 6 Service de Pneumologie et Allergologie, CHU, Reims, France; 7 Service de Pneumologie et Allergologie, CHU, Marseilles, France; 8 Service de Pneumologie et Allergologie, CHU, Bordeaux, France

**Keywords:** Childhood asthma, Environmental factors, Management, Early life, Under-diagnosis, Under-treatment

## Abstract

**Background:**

Under-diagnosis and under-treatment of childhood asthma were investigated in France using data collected during the 6 Cities Study, the French contribution to the International Study of Asthma and Allergies in Childhood.

**Methods:**

7,781 schoolchildren aged between 9 and 10 years underwent a medical visit including skin prick tests to common allergens and exercise test for Exercise-Induced Asthma (EIA) and their parents filled in a standardized questionnaire on asthma, management, treatment and potential risk factors.

**Results:**

903 children reported asthma (11.6%), 377 without a doctor’s diagnosis. Of the 526 participants with a diagnosis of asthma confirmed by a doctor (58.2%), 353 were treated and 76 were not treated during the year preceding the investigation despite their diagnosis. The information on the treatment was missing for the rest of individuals diagnosed with asthma (n = 97). Having a treatment was significantly associated with severe asthma and with the presence of other respiratory and allergic *stigmata* (atopic eczema, rhinitis, positive skin allergy tests, and EIA). In addition, having a treatment did not correspond to a good control of the disease. Similarly, children with asthma-like symptoms but without doctor-diagnosed asthma had asthma less well controlled than children with diagnosed asthma. They were also more exposed to passive smoking and traffic but had fewer pets. In contrast, diagnosed children reported more frequently a small weight at birth and a preterm birth.

**Conclusions:**

In France, childhood asthma is still under-diagnosed and under-treated and environmental factors play a role in these phenomena.

## Background

With about 300 million asthmatics worldwide, asthma has become very important in public health [1]. Furthermore, asthma is the most common disease in children with a prevalence varying between countries [2].

In France, nearly 4.15 million people were affected by this disease in 2006. Asthma affects all age groups but often occurs during childhood. The latest estimates indicate that 7 to 10% of children and 5 to 6% of adults are affected by an active form of asthmatic disease [3]. There are more boys under 10 years than girls of the same age with asthma. Mortality due to asthma mainly affects the elderly but also children under 15 years [4].

The burden of asthma is relevant for the health system in terms of both direct (hospitalization and treatment) and indirect costs (absenteeism in school for the children and at work for the parents) [5]. Globally, it was estimated that the costs associated with asthma exceeded those of tuberculosis and HIV/AIDS combined [4]. These costs could be reduced by a diagnosis and more appropriate control of the disease. However, asthma remains still under-diagnosed and uncontrolled to a large extent in spite of several recommendations [2-6]. In addition, there are no data allowing determining whether recommendations are followed. In most chronic diseases, patient’s education plays a major role in seeking care, and this should also follow in the management and control of asthma. Indeed, by acting on the diagnosis, care and patient education, it seems possible to reduce the burden associated with asthma [7].

Several studies have attempted to show why the asthma care was absent or delayed. They have all bowed down to the complexity of the topic. Actually, the management of the asthmatic disease remains a complex subject since it depends on a multitude of factors including the perception of parents, children, educators, and health professionals, as well as the availability of resources and adherence to treatment [8]. However, few factors have been studied in detail. In particular, very few investigations have considered the impact of physical environmental factors on asthma management.

Using data from the French 6 Cities Study conducted in a large population-based sample of primary schoolchildren residing in metropolitan France, we aimed at identifying risk factors associated with the presence or absence of asthma diagnosis and treatment as important constituents in the management of childhood asthma. The considered factors were individual, socio-demographic, clinical and environmental and included the early life window of exposure. The long-term purpose of our study is to understand what interventions are necessary to achieve optimal management of asthmatic disease.

## Methods

### Protocol and population

Through the 6 Cities Study, the French section of the second phase of ISAAC investigation conducted in France in 2000–1, 9,615 primary schoolchildren were invited in the six French cities (Bordeaux, Clermont-Ferrand, Créteil, Marseille, Strasbourg and Reims) to undergo clinical tests and their parents to complete a standardized medical questionnaire derived from the International ISAAC questionnaire [9]. The clinical tests, performed at school by qualified physicians, included a skin examination to detect atopic eczema, a test of bronchial hyperactivity to effort, and skin prick tests (SPT) to identify the existence of an allergic hypersensitivity.

### Questionnaire

The standardized questionnaire included sections on socio-demographic and risk factors, health (asthma, allergic rhinitis, eczema, allergies), management, use of care facilities, treatment, compliance, lifestyle, housing, early events of life. Children were also interviewed also on school absenteeism due to asthma. Details of the survey are presented elsewhere [10].

### Asthma definition and characterization

Exact standardized questions used to identify through the questionnaire children with asthma and to characterize them by the existence of a diagnosis or treatment were:

During the past 12 months, has your child (he/she) ever had wheezing or whistling in the chest at any time? (“YES” corresponded to have ever had wheezing or whistling)

Has your child ever had asthma? (“YES” corresponded to have ever had asthma)

Has your child ever been diagnosed with asthma by a doctor? (“YES” corresponded to have had a diagnosis of asthma).

Then, if the child was treated, the question was asked:

During the last 12 months, has your child (he/she) taken medication for wheezing or asthma also during or after physical effort? If the answer to this question was “YES”, the child was considered treated for his/her asthma. If the answer to this question was “NO”, the child was considered untreated.

The following three definitions of asthma (statistical variable) were used in our study:

· "Current asthma" as defined by report of wheezing or whistling in the chest in the last twelve months and ever asthma in life (dichotomous variable).

· "Asthma diagnosed by a physician" (dichotomous variable).

· "Treated asthma" (dichotomous variable).

In addition, to better characterize asthma the following characteristics were considered:

· Clinical severity of asthma according to GINA (http://www.gina.org), the number of crises during the past 12 months, the number of wheezing episodes that have awakened the child in the last 12 months, the number of asthma attacks, the number of crises that prevented him/her from speaking in the past 12 months, the number of wheezing episodes during or after exercise in the last 12 months, hospitalization during the last 12 months, and the number of school days missed in the last 12 months.

· Therapeutic intervention: asthma medication, asthma attacks prevention by parents, knowledge of medication to give for asthma attacks by parents, health care of the child's asthma, including prevention of asthma attacks, and the use of a peak flow-meter.

Allergic history and Exercise-Induced Asthma (EIA)

· Allergic sensitization was defined on the basis of positive skin allergy tests. The skin tests were performed by the SPT technique according to the ISAAC protocol with indoor and outdoor allergens *(*e.g. *Dermatophagoides pteronyssinus, Dermatophagoides farinae,* cat hair, *Alternaria,* cockroach, grass, etc.) and food allergens (e.g., the trophoallergenes, milk, fish, eggs, peanuts, etc.), with a positive and negative control to eliminate false positives and negatives [10]. Three variables were considered: the SPT positivity to indoor allergens, SPT positivity to outdoor allergens, and SPT positivity to trophoallergens.

· Exercise-induced Asthma (EIA) was assessed by measuring changes in peak expiratory flow before and after a running test lasting six minutes. We defined a test as positive when the peak expiratory flow rate fell to either less than 10% or less than 15%, respectively. In the analysis, we have used these two variables: BHR10 (bronchial hyper-responsiveness to effort with decreased lung function of 10%) and BHR15 (bronchial hyper-responsiveness to effort with decreased lung function of 15%).

· Allergic comorbidities: lifetime eczema, lifetime allergic rhinitis and other serious health problems.

### Studied co-factors

The following factors were considered as co-variables in the statistical models:

· Individual factors: the city (Bordeaux, Clermont-Ferrand, Créteil, Marseille, Strasbourg and Reims), sex, age in years, the Body Mass Index (BMI) defined by the ratio of weight to height squared in kg/m^2^, the heritability of asthma (i.e., maternal asthma) and number of siblings.

· Socio-demographic factors: ethnicity of the two parents, educational level of the two parents, paternal Socio-Economic-Status (SES) as defined by the classification of INSEE, i.e., the French National Institute for Statistics (e.g., farmers, employees,…), and marital status of the child’s parents and medical coverage (i.e., health insurance, mutual payment, free medical care, personal insurance, no welfare at all) of the family.

· Environmental condition at the period of the survey (e.g., smoking, pets, housing situation, tasks, moisture, exposure to traffic, etc.).

· Early life events, i.e., prematurity, birth weight, breastfeeding.

Dichotomous or categorical variables with the exception of BMI were considered in the analysis.

### Statistical and epidemiological analyses

The type of asthma (“diagnosed or not”, “treated or not”) was investigated with respect to child’s characteristics, clinical characteristics of asthma, co-morbidity, asthma management, environmental exposure at the period of the survey and early life events in order to identify factors that were associated with the absence of asthma diagnosis and treatment. The chi-square test was used to compare percentages between groups. Analysis of variance (ANOVA) was used to compare differences between continuous variables. Through a logistic regression analysis adjusting for age, sex, city and BMI, the factors associated with the different types of asthma were identified. Associations were expressed in terms of Odds Ratios (OR) and 95% confidence intervals (95% CI). The study on the use of asthma medications was restricted to children diagnosed with asthma, since not being diagnosed greatly diminished the chances of being treated for this disease. All statistical analyses were performed using SAS ® version 9.

## Results

**Figure 1:**
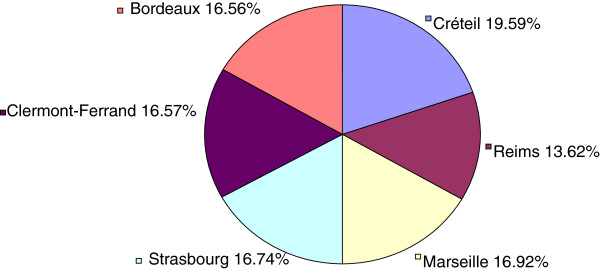
Geographical distribution in the 6 Cities Study (n = 7,781 participants).

Out of the 9,615 children in the classes of CM1 and CM2 recruited in 108 schools randomly selected in the 6 Cities, 7,798 were enrolled (Figure 1) for the study. They were aged between 9–10 years old [10]. The participation rate varied by city. We report here data for 903 children having suffered from asthma in the past 12 months (11.6%), including 526 (58.25%) participants who were diagnosed as asthmatic by a doctor. The remaining 377 children, while having asthma-like symptoms (e.g., wheezing or whistling in the chest in the last 12 months) did not receive a doctor’s diagnosis confirming the existence of asthma. From a total of 526 children diagnosed with asthma, 353 were treated for asthma and 76 were not treated. The information on the treatment was missing for the remainder of children diagnosed with asthma (n = 97).

Significant geographic differences were observed in the diagnosis and treatment of childhood asthma based on data (Table 1). The highest proportion of diagnosed asthma was seen in Créteil (19.39%), which is a city close to Paris, followed closely by Bordeaux (18.72%) and Marseilles (18.16%). Bordeaux (19.26%), Marseille (18.70%), and Créteil (18.70%) were the cities where children were the most treated ones; there were more untreated children in eastern France than elsewhere (18.42% in Reims and Strasbourg with 25.00%). After adjustment for potential confounders, most treated children were from Bordeaux and Strasbourg (OR) = 2.816, confidence interval 95% (95% CI): 1.121-7.075, p = 0.0436). The rate of untreated asthma out of diagnosed asthma varied from 4.7% in Bordeaux to 13.4% in Strasbourg (6.1% in Marseilles, 7.4% in Créteil and 8.8% in Clermont-Ferrand) (Table 1). Compared to girls, boys were at higher risk not only of reporting more asthma but also of suffering more of diagnosed and treated asthma (Table 2). Compared to the entire population, boys not only reported more asthma (60.35% vs. 39.64%) but also suffered more from undiagnosed and untreated asthma than girls (Table 2). Trends were found between asthma and ethnicity and living only with the mother.

**Table 1:** Geographic distribution of the children in the entire population-based sample and in the asthmatics

	**Entire sample**	**Asthma**				
		**All**	**Undiagnosed**	**Diagnosed**	**Diagnosed**	
	**N = 7,781**	**N = 903**	**N = 377**	**N = 526**	**Untreated N = 76**	**Treated N = 353**
N (%)						
Créteil	1,420	175	73	102	13	66
	(19.59)	(19.39)	(19.36)	(19.39)	(17.11)	(18.70)
Reims	987	116	40 (10.61)	76	14	50
	(13.62)	(12.85)		(14.45)	(18.42)	(14.16)
Marseille	1,226	164	69	95	10	66
	(16.92)	(18.16)	(13.12)	(18.06)	(13.16)	(18.70)
Strasbourg	1,213	142	59	83	19	55
	(16.74)	(15.73)	(15.65)	(15.78)	(25.00)	(15.58)
Clermont-Ferrand	1,201	137	62	75	12	48
	(16.57)	(15.17)	(16.45)	(14.26)	(15.79)	(13.60)
Bordeaux	1,200	169	74	95	8	68
	(16.56)	(18.72)	(19.63)	(18.06)	(10.53)	(19.26)

N, number;%, percentage.

**Table 2:** Characteristics of the children in the entire population-based sample and in the asthmatics

	**Entire sample**	**Asthma**				
		**All**^**(&)**^	**Undiagnosed**	**Diagnosed**^**(^)**^	**Diagnosed**	
	**N = 7,781**	**N = 903**	**N = 377**	**N = 526**	**Untreated N = 76**	**Treated**^**(*§)***^**N = 353**
**Variable**						
**Age** (year, mean ± DS)	10.40 ± 0.75	10.42 ± 0.74	10.40 ± 0.75	10.43 ± 0.74	10.43 ± 0.73	10.43 ± 0.71
**Gender**, N (%)						
Boys	3,872 (49.77)	545 (60.35)*	222 (58.89)	323 (61.41)	45 (59.21)	222 (62.89)
Girls	3,908 (50.23)	358 (39.65)	155 (41.11)	203 (38.59)	31 (40.79)	131 (37.11)
**BMI**, N (%)						
8 Kg/m^2^	4,751 (61.07)	529 (58.58)	224 (59.42)	305 (57.98)	45 (59.21)	199 (56.37)
18-25 Kg/m^2^	2,842 (36.53)	347 (38.43)	140 (37.14)	207 (39.35)	27 (35.53)	145 (41.08)
5 Kg/m^2^	187 (2.40)	27 (2.99)	13 (3.45)	14 (2.66)	4 (5.26)	9 (2.55)
**Mother’s asthma,** N (%)	521 (7.27)	151 (16.72)*	62 (16.45)	89 (16.92)	10 (13.16)	62 (17.56)
**Birth order, First,** N (%)	3,940 (55.01)	517 (57.32)	207 (55.05)	310 (58.94)	46 (60.53)	218 (61.76)
**Ethnic origin of the parents,** N (%)						
Metropolitan France	4,272 (54.90)	524 (78.80)*	205 (75.65)	319 (80.96)	49 (84.48)	215 (80.83)
Overseas Departments	98 (1.26)	14 (2.11)	4 (1.48)	10 (2.54)	2 (3.45)	8 (3.01)
South Europe	142 (1.82)	15 (1.78)	8 (2.95)	7 (1.78)	0	5 (1.88)
North Africa	461 (5.92)	57 (7.61)	27 (9.96)	30 (7.61)	4 (6.90)	21 (7.89)
Asia	169 (2.17)	24 (2.79)	13 (4.80)	11 (2.79)	1 (1.72)	7 (2.63)
Sub-Saharian Africa	137 (1.76)	11 (1.78)	4 (1.48)	7 (1.78)	0	5 (1.88)
Others	190 (2.44)	20 (2.54)	10 (3.69)	10 (2.54)	2 (3.45)	5 (1.88)
**Education**, N (%)						
Elementary	599 (9.10)	58 (7.00)	21 (6.03)	37 (7.71)	7 (10.14)	19 (5.88)
Secondary	2745 (41.70)	344 (41.55)	150 (43.10)	194 (40.42)	22 (31.88)	129 (39.94)
High School, College	3,103 (47.14)	404 (48.79)	165 (47.41)	239 (49.79)	38 (55.07)	169 (52.32)
Others	135 (2.05)	22 (2.66)	12 (3.45)	10 (2.08)	2 (2.90)	6 (1.86)
**Parental socio-economic status**, N (%)						
Artisan, own activity	339 (5.33)	39 (4.88)	15 (4.55)	24 (5.11)	2 (2.99)	17 (5.36)
Liberal profession	1,525 (23.97)	185 (23.13)	75 (22.73)	110 (23.40)	14 (20.90)	76 (23.97)
Intermediate profession	1,393 (21.89)	195 (24.38)	73 (22.12)	122 (25.96)	25 (37.31)	83 (26.18)
Employees	1856 (29.17)	228 (28.50)	105 (31.82)	123 (26.17)	11 (16.42)	79 (24.92)
Blue collars	871 (13.69)	98 (12.25)	38 (11.52)	60 (12.77)	12 (17.91)	39 (12.30)*
Farmer, retired, inactive	379 (5.96)	55 (6.88)	24 (7.27)	31 (6.60)	3 (4.48)	23 (7.26)
**Medical coverage**, N (%)						
Social Security	6,846 (87.99)	864 (95.68)	363 (96.29)	501 (95.25)	69 (90.79)	341 (96.60)*
Complementary insurance	5,813 (74.71)	733 (81.17)*	305 (80.90)	428 (81.37)	63 (82.89)	285 (80.74)
Free medical care	514 (6.61)	74 (8.19)	37 (9.81)	37 (7.03)	8 (10.53)	24 (6.52)
Personal insurance	990 (12.72)	106 (11.74)	43 (11.41)	63 (11.98)	14 (18.42)	37 (10.48)
No protection	12 (0.15)	5 (0.55)	1 (0.27)	4 (0.76)	0	2 (0.57)
**Type of family**, N (%)						
Live with his/her 2 parents	5,140 (72.24)	584 (64.82)*	236 (62.93)	348 (66.16)	51 (67.10)	237 (67.14)
Live with his/her mother only	1,487 (20.90)	234 (25.97)*	101 (26.93)	133 (25.29)	17 (22.37)	87 (24.65)
Live with his/her father only	179 (2.52)	23 (2.55)	12 (3.20)	11 (2.09)	1 (1.32)	7 (1.98)

N, number;%, percentage; NS, not significant; **(**&**),** p-value between asthmatics and the rest of the population; **(^),** p-value between diagnosed and un-diagnosed asthma; (§), p-value between treated and untreated asthma.

*, p < 0.05.

### Diagnosed vs. undiagnosed asthma

Comparing the presence and the absence of a doctor’s diagnosis of asthma in asthmatics, diagnosed asthma was found to be less frequent when parents had low education or a foreign origin (North Africa or Asia). Indeed, asthma was most frequently diagnosed among children of French origin (metropolitan France and overseas territories) (Table 2). No significant association was observed with other socio-demographic and individual factors (Table 2).

As expected, the treatment varied according to the presence or the absence of the diagnosis of asthma (Table 3). Among the diagnosed asthmatics, bronchodilators were the drug class most often used, with 59.32% of children who had taken at least one drug in this class (Table 3), followed by inhaled corticosteroids (39.73%). In the sample of the children not diagnosed with asthma, there were children who took medication to improve their breathing that required a prescription even in the absence of a diagnosis of asthma (7.16% were taking bronchodilators and 11.94% were taking inhaled corticosteroids) (Table 3).

**Table 3:** Therapeutic intervention in asthmatics of the 6 Cities Study (n = 903 out of 7,781 participants)

**Asthma medication**	**Undiagnosed Asthma N = 377**	**Diagnosed Asthma N = 526**	**p-value**
**Antihistamines h1**, N (%)	11 (2.92)	65 (12.36)	0.01
**Inhaled Corticosteroids**, N (%)	27 (7.16)	209 (39.73)	0.01
**Bronchodilators**, N (%)	45 (11.94)	312 (59.32)	0.01
**Other antihistamines**, N (%)	4 (1.06)	42 (7.98)	0.01
**Nasal medications**, N (%)	0	9 (1.71)	NS
**Medication against eczema**, N (%)	0	3 (0.57)	NS
**Other medications**, N (%)	6 (1.59)	12 (2.28)	NS

N, number; NS, not significant;%, percentage.

The clinical comparison between diagnosed and undiagnosed asthma showed that among the undiagnosed asthmatics, 3.98% (vs. 14.07% among diagnosed) were in the GINA level 2 and 5. 85% (vs. 17.87% among diagnosed) in level 3 (Table 4). In the last 12 months, 66.22% of undiagnosed asthma cases had had one to three crises (vs. 55.14% among diagnosed), 6.54% of children woke up on one or more nights in a week (vs. 10.29% among diagnosed) due to wheezing, 4.05% of children had a severe attack that had prevented him/her from speaking (vs. 10.14% among diagnosed), and 28.87% had missed one school day at least (vs. 39.10% among diagnosed). In terms of comorbidities, diagnosed asthmatics were more often diagnosed with eczema (47.60% vs. 33.43%) and lifetime allergic rhinitis (66.86% vs. 54.44%) than the undiagnosed ones (Table 5). In addition, the management of the disease was less important in the undiagnosed asthma group as shown by lower percentages of medication compliance, attacks prevention, medication use during asthma attacks, asthma management and peak flow use in this group (Table 6). Undiagnosed asthmatic children were more exposed to maternal smoking and traffic (living near a bus stop), but had fewer pets than the diagnosed children (Table 7). In contrast, diagnosed children had a past history of low birth weight and preterm birth more often than the undiagnosed ones (Table 8).

**Table 4:** Clinical characteristics of asthma (N = 903)

	***Undiagnosed Asthma N = 377***	***Diagnosed Asthma (^) N = 526***	***Diagnosed Asthma***	
			**Untreated N = 76**	**Treated(§) N = 353**
**Asthma severity according to GINA**, N (%)		***		***
level 1	340 (90.19)	358 (68.06)	70 (92.11)	202 (57.22)
level 2	15 (3.98)	74 (14.07)	2 (2.93)	71 (20.11)
level 3	22 (5.85)	94 (17.87)	4 (5.26)	80 (22.66)
**Number of attacks during the last 12 months,** N (%)		**		***
None	53 (23.56)	56 (15.14)	17 (58.62)	21 (6.95)
1 to 3 times	149 (66.22)	204 (55.14)	12 (41.38)	177 (58.61)
4 to 12 times	18 (8.00)	86 (23.24)	0	81 (26.82)
More than 12 times	5 (2.22)	24 (6.46)	0	23 (7.62)
**Sleep disturbed due to wheezing**, N (%)				*
Never	162 (72.70)	200 (57.14)	21 (80.77)	157 (53.58)
Less than a night per week	38 (17.76)	114 (32.57)	3 (11.54)	105 (35.84)
One or more nights per week	14 (6.54)	36 (10.29)	2 (7.69)	31 (10.58)
**Asthma attacks**, N (%)				***
1 or more crises per month	13 (13.13)	108 (25.71)	1 (1.64)	101 (31.08)
Less than a crisis per month	24 (24.24)	107 (25.48)	5 (8.20)	100 (30.70)
Less than a crisis by year	27 (27.27)	83 (19.76)	11 (18.03)	70 (21.54)
Attacks have disappeared	35 (35.35)	122 (29.05)	44 (72.13)	54 (16.62)
**Wheezing ever been severe enough to limit your (child’s) speech in the last 12 months**, N (%)	9 (4.05)	37 (10.14)	1 (3.70)	35 (11.59)
**Wheezing during or after effort in the last 12 months**, N (%)	106 (29.12)	217 (42.27)	6 (7.89)	200 (58.48%)***
**Hospitalization in the last 12 months**, N (%)	2 (2.06)	32 (8.94)	0	30 (10.68)
**Missed school days**, N (%)				**
None	69 (71.13)	215 (60.91)	49 (90.74)	152 (53.71)
1-5 days	18 (18.56)	87 (24.65)	2 (3.70)	83 (29.33)
6-10 days	6 (6.19)	36 (10.20)	3 (5.56)	33 (11.66)
More then 11 days	4 (4.12)	15 (4.25)	0	15 (5.30)

N, number; NS, not significant,%, percentage.

**(^),** p-value between diagnosed and undiagnosed asthma.

(§), p-value between treated and untreated asthma.

*, p < 0.05.

**, p < 0.01.

***p < 0.001.

**Table 5:** Comorbidity in the studied population-based sample and the asthmatics

	**Entire sample N = 7,781**	**Asthmatics N = 903**	**Undiagnosed Asthma N = 377**	**Diagnosed Asthma N = 526**	**Diagnosed Asthma**		**p-value**^**§**^
					**Untreated N = 76**	**Treated N = 353**	
**Lifetime eczema**, N (%)	1,715 (25.22)	359 (41.65)	121 (33.43)	238 (47.60)	27 (36.00)	175 (52.24)	0.0110
**Lifetime allergic rhinitis**, N (%)	1,998 (29.28)	537 (61.72)	196 (54.44)	341 (66.86)	41(55.41)	242 (70.76)	0.0102
**Severe health problem**, N (%)	287 (4.28)	52 (6.30)	17 (5.04)	34 (7.16)	5 (6.85)	23 (7.12)	
**SPT + to indoor allergens**, N (%)	1,409 (20.95)	342 (45.60)	119 (37.07)	223 (51.98)	20	167	<0.0001
					(30.30)	(60.29)	
**SPT + to outdoor allergens**, N (%)	824 (12.25)	184 (24.53)	56 (17.45)	128 (29.84)	12	95	0.0111
					(18.18)	(34.30)	
**SPT + to food allergens**, N (%)	138 (2.05)	60 (4.00)	6 (1.87)	24 (5.59)	2 (3.03)		
**BRH10,** N (%)	610 (8.95)	140 (17.95)	55 (16.72)	85 (18.85)	6 (8.82)	69 (23.31)	0.0077
**BRH15,** N (%)	227 (3.33)	85 (10.90)	28 (8.51)	57 (12.64)	2 (2.94)	50 (16.89)	0.0030

HBR10, bronchial hyperactivity to effort with respiratory function decreasing of 10%; HBR15, bronchial hyperactivity to effort with respiratory function decreasing of 15%; SPT +, skin prick test positivity; N, number; NS, not significant;%, percentage.

**(**&**),**p-value between asthmatics and the rest of the population.

**(^),** p-value between diagnosed and undiagnosed asthma.

(§); p-value between treated and untreated asthma.

*, p < 0.05.

**Table 6:** Management of asthma by the asthmatics (N = 903)

	**Undiagnosed Asthma N = 377**	**Diagnosed Asthma N = 526**	**Diagnosed Asthma**		**p-value**^**§**^
			**Untreated N = 76**	**Treated N = 353**	
**Medication compliance**, N (%)					
All treatment	34 (73.91)	262 (72.58)	6 (50.00)	243 (73.41)	<0.0001
Most of the treatment	6 (13.04)	66 (18.28)	1 (8.33)	62 (18.73)	
A part of the treatment	6 (13.04)	21 (5.82)	0	20 (6.04)	
None	0 (0.00)	8 (2.22)	4 (33.33)	3 (0.91)	
**Attacks prevention,** N (%)	33 (31.43)	281 (62.86)	31 (45.59)	223 (67.58)	0.0006
**Medication during asthma attacks**, N (%)	68 (64.15)	423 (91.36)	59 (84.29)	325 (94.20)	0.0040
**Management of asthma by the child**, N (%)	21 (23.08)	300 (66.52)	23 (32.39)	258 (77.48)	<0.0001
**Peak flow-meter use**, N (%)	0 (0.00)	29 (6.25)	2 (2.99)	25 (7.20)	0.2003

N, number; NS, not significant;%, percentage.

**(**&**),** p-value between asthmatics and the rest of the population.

**(^),** p-value between diagnosed and undiagnosed asthma.

(§), p-value between treated and untreated asthma.

*, p < 0.05.

**Table 7:** Environmental exposure of the studied population-based sample and in the asthmatics

	**Entire sample N = 7,781**	**Asthmatics N = 903**	**Undiagnosed Asthma N = 377**	**Diagnosed Asthma N = 526**	**Diagnosed Asthma**		**p-value**^**§**^
					**Untreated N = 76**	**Treated N = 353**	
**Paternal smoking**, N (%)	1,958 (27.31)	251 (27.80)	113 (29.97)	138 (26.24)	17 (22.37)	97 (27.48)	NS
**Maternal smoking**, N (%)	2,060 (28.73)	294 (32.56)	136 (36.07)	158 (30.04)	20 (26.32)	110 (31.16)(	NS
**Living in**, N (%)							NS
Town	4,625 (67.48)	593 (68.79)	258 (71.67)	335 (66.73)	47 (68.12)	225 (66.37)	
Suburb of a town	1,633 (23.83)	199 (23.09)	76 (21.11)	123 (24.50)	14 (20.29)	90 (26.55)	
Village	369 (5.38)	39 (4.52)	15 (4.17)	24 (4.78)	4 (5.80)	13 (3.83)	
Isolated house, farm	227 (3.33)	31 (3.60)	11 (3.06)	20 (3.98)	4 (5.80)	11 (3.24)	
**House built**, N (%)							
Before 1945	1,444 (21.44)	202 (23.71)	92 (26.44)	110 (21.83)	12 (17.14)	77 (22.65)	NS
Between 1945-1960	832 (12.35)	86 (10.09)	32 (9.20)	54 (10.71)	9 (12.86)	33 (9.71)	
After 1960	3,150 (46.76)	387 (45.42)	144 (41.38)	243 (48.21)	35 (50.00)	164 (48.24)	
Don’t know	1,310 (19.45)	177 (20.77)	80 (22.99)	97 (19.25)	14 (20.00)	66 (19.41)	
**Living near bus stops** ,N (%)	2,781 (41.07)	358 (41.68)	167 (47.18)	191 (37.82)	17 (23.94)	137 (40.41)	0,0092
**Gas cooking, heater with evacuation**, N (%)	3,340 (49.10)	424 (49.19)	167 (47.04)	257 (50.69)	35 (48.61)	167 (49.26)	NS
**Conditioned air**, N (%)	400 (5.84)	56 (6.50)	23 (6.44)	33 (6.53)	4 (5.41)	18 (5.31)	NS
**Cracked painting at home**, N (%)	790 (11.45)	119 (13.63)	48 (13.26)	71 (13.89)	10 (13.51)	45 (13.12)	NS
**Condensation**, N (%)	1,225 (18.10)	172 (20.02)	75 (20.89)	97 (19.40)	13 (18.60)	69 (20.60)	NS
**Water leaks**, N (%)	551 (7.97)	74 (8.47)	30 (8.26)	44 (8.61)	3 (4.05)	30 (8.72)	NS
**Moulds**, N (%)	1,176 (17.02)	166 (19.10)	71 (19.72)	95 (18.66)	17 (22.97)	63 (18.48)	NS
**Pets**, N (%)	3,643 (50.81)	406 (44.96)	169 (41.63)	237 (58.37)	32 (42.11)	160 (45.33)	NS

N, number; NS, not significant;%, percentage.

(&**);** p-value between asthmatics and the rest of the population.

**(^),** p-value between diagnosed and un-diagnosed asthma.

(§), p-value between treated and untreated asthma.

*:p < 0.05.

**Table 8:** Early life events in the studied population-based sample and in asthmatics

	**Entire sample N = 7,781**	**Asthmatics N = 903**	**Undiagnosed Asthma N = 377**	**Diagnosed Asthma N = 526**	**Diagnosed Asthma**		**p-value**^§^
					**Untreated N = 76**	**Treated N = 353**	
**Birth weight**, N (%)							NS
<2.5 kg	402 (6.16)	61 (7.61)	29 (8.81)	32 (6.77)	2 (2.90)	22 (6.94)	
2.5 kg −3.5 kg	4,191 (64.25)	512 (63.84)	224 (68.09)	288 (60.89)	44 (63.77)	192 (60.57)	
3.5 kg<	1,930 (29.59)	229 (28.55)	76 (23.10)	153 (32.35)	23 (33.33)	103 (32.49)	
**Pre-term birth**, N (%)	4,829 (25.88)	264 (32.51)	117 (35.35)	147 (30.56)	24 (31.58)	93 (26.35)	NS
**Breast-fed child**, N (%)	3,709 (54.36)	460 (53.74)	197 (56.29)	263 (51.98)	33 (45.21)	186 (55.36)	0.1150

HBR10, bronchial hyperactivity to effort with respiratory function decreasing of 10%; HBR15, bronchial hyperactivity to effort with respiratory function decreasing of 15%, NS, not significant; SPT +, skin prick test positivity.

(&**),** p-value between asthmatics and the rest of the population.

**(^),** p-value between diagnosed and undiagnosed asthma.

(§), p-value between treated and untreated asthma.

*, p < 0.05.

### Treated vs. untreated asthma

Even among the children not diagnosed with asthma there were children who took medication (Table 3). There was no statistical significant difference between treated and untreated asthmatics with respect to gender, age and BMI (Table 2). Asthma was most frequently treated among children of French origin (metropolitan France and overseas territories). There were fewer treated asthma cases when the educational level of parents was limited to primary education (9.10% vs. 5.88%) and a greater proportion of treated asthma cases for higher levels of parental education (47.14% vs. 52.32%, when compared to the study general population). In addition, medical coverage significantly influenced the treatment of children since those covered by the social security system were more treated compared to the others (p = 0.03). The clinical condition (Table 4) was certainly the most convincing for getting a treatment among the asthma cases. Asthmatics with untreated asthma in most case were on level 1 according to asthma severity level in GINA (92.11% vs. 57.22%). Children whose clinical condition was more severe (levels 2 and 3) were more often treated than those of level 1 (level 2: OR = 11.619, 95% CI: 2.738 – 49.311, p = 0.0009; and level 3: OR = 6.680, 95% CI: 2.330 – 19.065, p = 0.0004). Nevertheless, there were still 2.93% of diagnosed asthmatics who were not treated in the level 2 and 5.26% in level 3. It was observed that the number of attacks in the last year and the number of attacks that have awakened the child were highly related to treatment, with untreated asthma having fewer crises than treated asthma.

The treated children also had more hospitalizations (no hospitalization: 100.00% among non-treated vs. 89.32% among those treated) and missed more days at school (no school day missed; 90.74% in untreated vs. 53.71% for treated). Comorbidities (Table 5) were also factors that were revealing in our study. Being treated for asthma was strongly associated with comorbidities, such as lifetime eczema (OR = 2.206, 95% CI: 1.269 – 3.835, p = 0.0050), allergic rhinitis (OR = 3.055, 95% CI: 1.310 – 7.122, p = 0.0097) and allergic sensitization to indoor allergens (OR = 3.691, 95% CI: 2.024 – 6.734, p = <0.0001), allergic sensitization to outdoor allergens (OR = 2.458, 95% CI: 1.215 – 4.970, p = 0.0123), BRH10% (OR = 2.854, 95% CI: 1.157 – 7.040, p = 0.0228) and BRH15% (OR = 5.950, 95% CI: 1.389 – 25.482, p = 0.0163). The knowledge of the management of asthma was more frequent in treated children compared to the others (Table 6). Regarding the environment at the period of the survey (Table 7), living near a bus stop was the only factor found to be treatment-related. Among the events of early life, none influenced the treatment of asthma (Table 8). However, children who were breastfed were more frequently treated although only at a borderline significance (p = 0.11).

All of the factors identified as statistically related to the treatment for asthma in the univariate analysis remained statistically significant after adjustment for potential confounding factors (Table 9).

**Table 9:** Factors related to asthma treatment after adjustment on age sex, center and BMI in the asthmatics

	**Odds Ratio**	**Confidence interval 95%**	**p-value**
			
**Centres**			
Bordeaux vs. Strasbourg	2.816	1.121–7.075	0.0436
**Asthma severity**			
Level 2 vs. Level 1	11.619	2.738–49.311	0.0009
Level 3 vs. Level 1	6.680	2.330–19.065	0.0004
**Number of attacks**			
1 to 3 attacks vs. no crisis	10.202	3.843–27.084	<0.0001
**Night awakening**			
Less than one crisis/week vs. never	4.029	1.139–14.253	<0.0307
**Asthma attacks**			
Less than one attack per year vs. crises have disappeared	4.428	2.017–9.722	0.0002
Less than one attack per month vs. crises have disappeared	16.652	5.948–46.616	<0.0001
1 or more attacks per month vs. crises have disappeared	70.728	9.414–531.388	<0.0001
**Wheezing after an exercise**	17.384	7.165–42.178	<0.0001
**Missed school days**			
One day and more vs. none	11.480	2.650–49.734	0.0011
**Lifetime eczema**	2.206	1.269–3.835	0.0050
**Lifetime allergic rhinitis**	3.055	1.310–7.122	0.0097
**SPT Positivity to indoor allergens**	3.691	2.024–6.734	<0.0001
**SPT Positivity to outdoor allergens**	2.458	1.215–4.970	0.0123
**HBR10**	2.854	1.157–7.040	0.0228
**HBR15**	5.950	1.389–25.482	0.0163
**Medication compliance**			
Most of the treatment vs. nothing	37.648	1.742–813.854	0.0207
All treatment vs. nothing	36.005	3.169–409.117	0.0039
**Attacks prevention**	2.311	1.317–4.054	0.0035
**Medication during asthma attacks**	2.857	1.222–6.680	0.0154
**Management of asthma by the child**	6.697	3.702–12.113	<0.0001
**Living near a bus stop**	2.423	1.298–4.522	0.0055

HBR10, bronchial hyperactivity to effort with respiratory function decreasing of 10%; HBR15, bronchial hyperactivity to effort with respiratory function decreasing of 15%; SPT +, skin prick test positivity.

## Discussion

This study constitutes a preliminary attempt to identify which factors are associated with the under-diagnosis and the under-treatment of childhood asthma in Metropolitan France. Our population-based sample comprised 903 asthmatic children at the period of the survey, 58% of whom had been diagnosed as asthmatic by a doctor. Only 67% of children with a diagnosis of asthma were treated for their condition. After adjustment for potential confounding factors, the treatment was significantly related to two parameters, clinical status and comorbidities, with the treated children presenting more severe forms of asthma and more allergic comorbidities. Of note, there were children in our population having been diagnosed by a physician as asthmatics that were not treated for their condition. Essentially our findings highlight the need to identify populations of asthmatics most at risk of not being diagnosed and treated for their conditions. This in a context in which there is evidence that treatment is one effective way to control asthma [2] together with education, parental education in the case of young children [11]. Data show that the diagnosis constitutes a first necessary step in getting a treatment.

In our investigation, comorbidities, such as eczema, rhinitis, and allergic sensitization increased the likelihood of treatment, as asthma severity did. The treatment in our population was related to a greater severity, more frequent asthma attacks, more frequent awakenings due to wheezing, and a higher number of school days missed thus confirming previous data [12]. Some children with asthma in our population were at level 3 of GINA classification, despite their treatment. Previous observations have shown that patients severely affected by asthma did not tend to follow the treatment entirely, thus remaining uncontrolled with asthma [13]. However, children with treated asthma knew better how to control their asthma and medications to take in case of crisis attacks in our population. This seems plausible if there was a contact of the child with a health professional that led to a better understanding of his/her asthmatic condition. The GINA report has even proposed a patient-physician partnership for a better prevention of asthma. Another argument in favor of under-treatment of asthma in our population is provided by the type of treatment used. There was a majority of children taking bronchodilators for both attacks prevention and/or attacks, which does not seem appropriate in the light of existing data [14].

In this same population-based sample, we had observed an increased risk of asthma and allergies in children living close to areas with elevated concentrations of traffic-related pollutants [15]. We also found that almost a third of the children had lived all their life at the same address and thereafter were exposed through their life to elevated levels of traffic-related air pollutants. Proximity of an asthmatic’s house to a bus stop was an indicator whether he/she had more severe asthma and was more often treated. Several studies have shown that urban traffic is associated with increased respiratory symptoms and a greater use of treatment probably because of the action of traffic-related pollutants [16].

Our study also investigated the characteristics of the asthmatic population for whom no diagnosis was made. With one third of asthmatics-or 377 individuals-undiagnosed, our work shows that childhood asthma is still an under-diagnosed condition in France and that this situation can be detrimental. Indeed, children with asthma symptoms without a diagnosis had a less well controlled asthma than children with diagnosed asthma in our population. Under-diagnosis of asthma could be due to the reduced use of health care in some subjects, but also secondary to the use of differential diagnoses (e.g., bronchitis, asthmatic bronchitis, etc.) from the doctor since some of these children were taking drugs to improve their breathing [17]. Undiagnosed asthmatics in the 6 cities showed allergic sensitization and bronchial hyper-responsiveness (i.e., bronchospasm during exercise), known to be risk factors for asthma. The links between asthma and other allergies are already known, but have rarely been connected with the under-diagnosis and under-treatment of asthma. Allergic rhinitis and eczema are associated with more severe asthma [18]. In addition, allergic rhinitis is also associated with a sub-diagnosis and under-treatment [19].

We also observed that the perception and the assumption of disease were lower in the undiagnosed asthmatic population. When we looked at the asthma medication in people who were not diagnosed, we found that there was a significant decrease in appropriate treatments. Our study also show that children with symptomatic asthma but no diagnosis, had less well-controlled asthma than children with diagnosed asthma, since their quality of life determined by nocturnal awakenings and truancy was not optimal [20]. In previous studies, it was shown that the health implications could be significant in asthmatics without a proper diagnosis, because they were less treated than diagnosed asthmatics [21]: they missed more days of school because of wheezing, they limited their physical activity, and their sleep was more disrupted [22].

Our study presents some weaknesses but also many strengths. The major weakness is that at any step the study of the 6 cities was intended as a pharmaco-epidemiological investigation, which reduces the information regarding the specific treatment(s) taken by children. Our study would have taken benefit from more details on the dose, start and end of treatment, and frequency of administration. However, the questionnaire contained a section asking for details on asthma and this allowed collecting information on asthma treatment and compliance and management. Another bias might arise from the fact that parents of children reported treatments and symptoms. No objective data on treatment and asthma (for example, using the medical record) were available in the survey. In addition, it would have been interesting to have additional data on what factors predispose parents not to treat their children. Another aspect that may engender biases is that only one year before the survey was considered. Finally, data collection was retrospective, which raises the possibility of recall bias. All these biases preclude a more accurate corroboration of data and push for caution in interpreting the results. However, the selection of the population and the use of standardized protocol and instruments in our survey constitute an added value. In addition, data are original for France where data management is still limited to some surveys that did not specifically target children (http://www.irdes.fr).

## Conclusions

In conclusion, we examined the under-diagnosis of childhood asthma and the use of asthma medication in a population-based sample of schoolchildren. With one-third of children with symptoms suggestive of asthma who had no diagnosis of asthma, it remains a disease not controlled in our sample that was representative. Furthermore, the use of medication against asthma seems inadequate in the considered sample both qualitatively (the drugs were not always appropriate) and quantitatively (some asthmatics were not treated at all). Similar studies should help improving the level of intervention and treatment practices in France. This study constitutes a preliminary attempt to identify the characteristics of children undiagnosed and/or untreated for asthma compared to children diagnosed and/or treated for asthma in Metropolitan France, so as to emphasize the role of the diagnosis, the treatment, the education, and the consensus in the progression of the management of the asthmatic disease during childhood.

## Competing interests

The authors declare that they have no competing interests.

## Author’s contribution

IAM is the PI of the Six Cities Study. All the authors but CS conducted the survey and collected the data. IAM and CS conducted the analysis and wrote the manuscript. All the authors read and approved the final manuscript
